# [Retracted] Sevoflurane attenuates brain damage through inhibiting autophagy and apoptosis in cerebral ischemia-reperfusion rats 

**DOI:** 10.3892/mmr.2025.13656

**Published:** 2025-08-18

**Authors:** Cun-Xian Shi, Jin Jin, Xue-Qin Wang, Teng Song, Guang-Hong Li, Ke-Zhong Li, Jia-Hai Ma

Mol Med Rep 21: 123–130, 2020; DOI: 10.3892/mmr.2019.10832

Following the publication of this paper, it was drawn to the Editor's attention by a concerned reader that, regarding the immunohistochemistry experiments shown in Fig. 4A, the ‘Tak-242’ and the ‘Se+Tak-242’ panels appeared to show an overlapping section of data, and for the western blot data shown in Figs. 6 and 7, the β-actin data in Fig. 6A were strikingly similar to the NF-κB data in Fig. 7A, such that data which were intended to show the results from differently performed experiments had apparently been derived from the same original sources. Following an independent analysis of the data in the Editorial Office, a small overlapping section of data was also identified comparing the ‘Tak-242’ and ‘Se’ panels for the cellular morphological images in Fig. 5A, and the western blots featured in Fig. 6A were found to have subsequently reappeared in a paper written by different authors at different research institutes in the journal *Drug Design, Development and Therapy*.

In view of the various instances of data duplication identified in this paper, and the reappearance of certain of the data in a subsequent article, the Editor of *Molecular Medicine Reports* has decided that this paper should be retracted from the Journal on account of a lack of confidence in the presented data. The authors were asked for an explanation to account for these concerns, but the Editorial Office did not receive a satisfactory reply. The Editor apologizes to the readership for any inconvenience caused.

